# Spontaneous resolution of hepatic artery aneurysms due to segmental arterial mediolysis

**DOI:** 10.1002/ccr3.9238

**Published:** 2024-08-05

**Authors:** Serhat Kaya, Gamze Akkuzu, Ali Dablan, Cemal Bes, İlgin Özden, Aytül Hande Yardımcı

**Affiliations:** ^1^ Department of Radiology Başakşehir Çam&Sakura City Hospital Istanbul Turkey; ^2^ Department of Internal Medicine (Rheumatology Unit) Başakşehir Çam&Sakura City Hospital Istanbul Turkey; ^3^ Department of Radiology General Surgery (Liver Transplantation & Hepatopancreatobiliary Surgery Unit) Başakşehir Çam&Sakura City Hospital Istanbul Turkey

**Keywords:** aneurysm, computed tomography, segmental artery mediolysis, spontaneous resolution

## Abstract

In asymptomatic patients with lesions in SAM measuring <3 cm, conservative monitoring is an appropriate option, with the anticipation of uncomplicated recovery in some cases.

## 1 CASE

**FIGURE 1:**
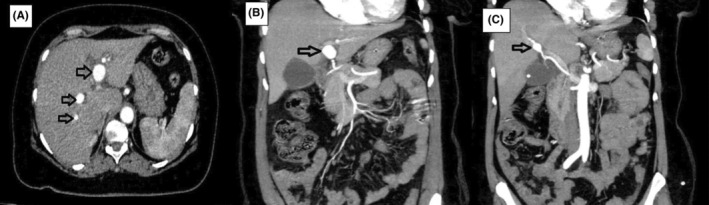
(A) Black arrows indicate right and left hepatic artery aneurysms on axial (A) and coronal MIP images (B, C) respectively.

A 55‐year‐old woman had been admitted to the emergency department in April 2022 for right upper abdominal quadrant pain. A contrast‐ enhanced computed tomography (CT) scan revealed three aneurysms in the branches of the hepatic artery: left (25 mm) right anterior (8 mm) and right posterior (4 mm) (Figure 1); the gallbladder was filled with stones. She had an ischemic cerebrovascular accident 20 years prior and had been diagnosed with Sjögren's syndrome (eye dryness and anti‐SSA antibody positivity) 16 years ago but had gone untreated for the last 6 years except for irregular, unsupervised, low‐dose acetylsalicyclic acid use.

**FIGURE 2:**
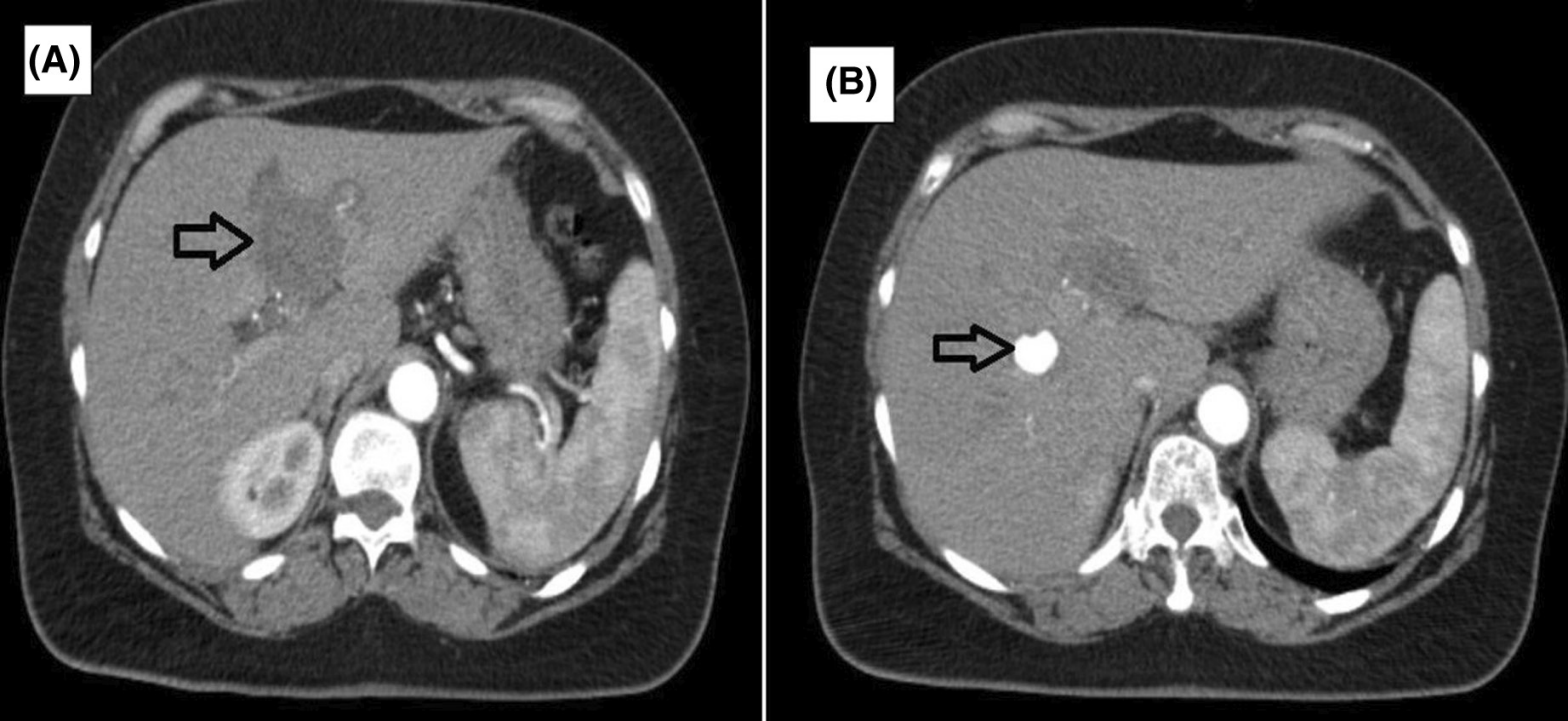
The black arrow shows the thrombosed aneurysm in the left hepatic artery (A), and the aneurysm in the right hepatic artery (B).

**FIGURE 3:**
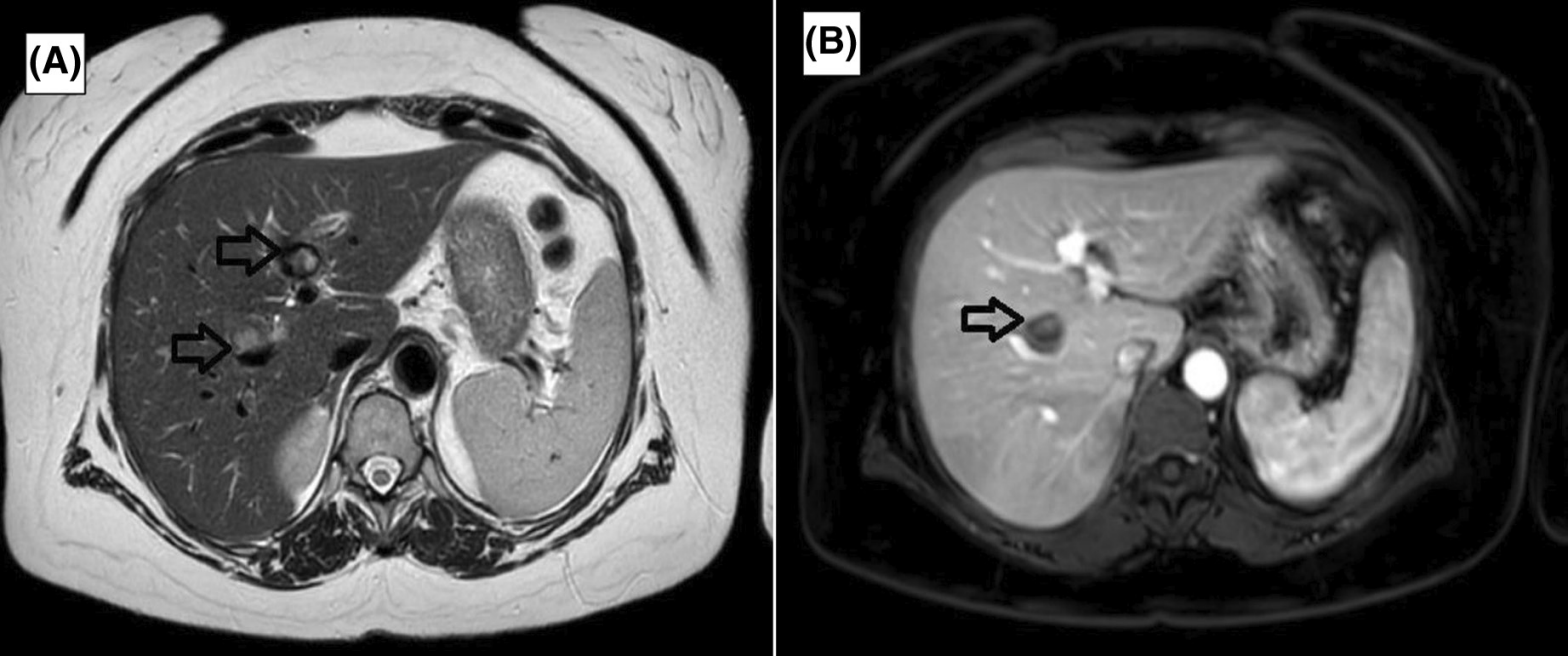
The black arrows show the thrombosed aneurysms in the right and left hepatic arteries on the axial T2W (A) and the contrast‐enhanced T1W magnetic resonance images (B).

She was examined by the rheumatology department on an outpatient basis and eventually referred to the liver transplantation & hepatopancreatobiliary surgery unit in July 2022. Follow up triphasic CT angiography showed that the aneurysm in the left branch had thrombosed spontaneously and the ones in the right anterior (10 mm) and posterior (6 mm) branches had slightly increased in size (Figure 2). The diagnosis of segmental arterial mediolysis was established based on the concordance of clinical, laboratory, and imaging findings according to the criteria determined by Kalva et al.^1^ MRI angiography in September 2022 showed that the aneurysms in the right hepatic artery branches had also thrombosed spontaneously (Figure 3).

**FIGURE 4:**
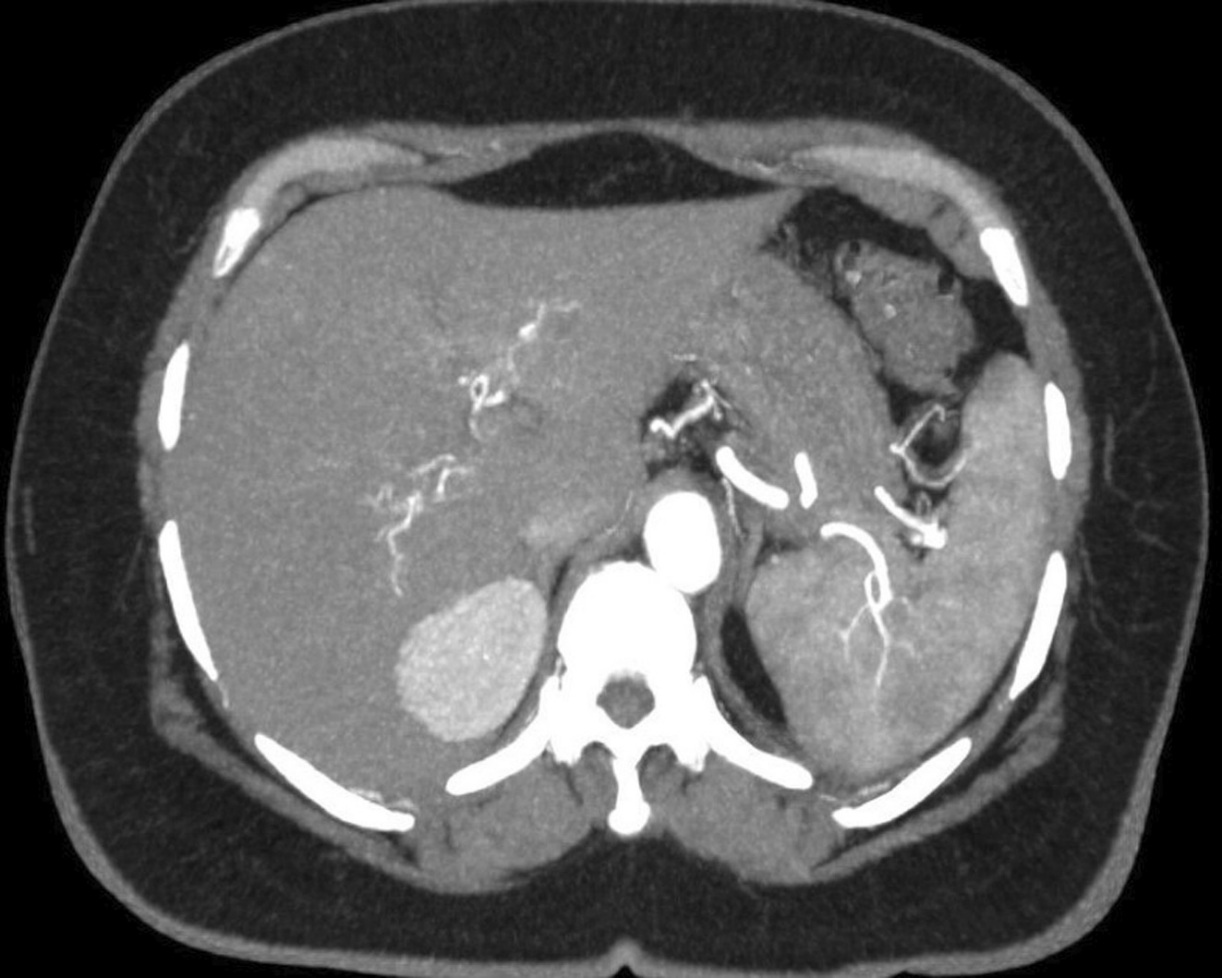
In the final image of the contrast‐enhanced aortic CT angiography, all aneurysms had spontaneously regressed and no vascular pathology was observed.

Digital subtraction angiography of the renal and hepatic arteries was conducted to investigate the presence of small aneurysms that might have been missed on cross‐sectional imaging and no abnormal findings were observed. Follow up the contrast‐enhanced abdominal CT in March 2023 and MRI in July 2023 showed no new aneurysms (Figure 4).

In the single center study by Naidu et al, 19 of the 97 (out of 111) followed patients (20%) showed disease progression (in the form of aggravation in the existing lesions or appearance of a new aneurysm); stability was observed in 42 patients (43%), resolution only in 18 patients (18.5%), and a mix of stability and resolution in 18 (18.5%).^2^ In the multicenter study by Shimohira et al, data on 123 aneuryms (31 ruptured) in 45 patients were reported. Twenty of the unruptured aneurysms were treated by transarterial embolization and one by resection. Seventy aneurysms in 30 evaluable patients were analyzed for natural history. No changes were observed in 45 aneurysms while nine showed a reduction in size and 13 disappeared; however, three new aneurysms developed during follow up. An interesting observation was the eventual disappearance of the second aneurysm in a patient who underwent emergency treatment for rupture. The median(range) period between the first examination and the appearance of morphologic changes was 10 months (2 days–99 months), disappearance of morphologic changes was 6 months (4 days–9 months), and reduction in morphologic changes was 2 months (1–99 months).^3^

In conclusion, although hepatic artery aneurysms in SAM may present with serious complications, conservative follow up is a feasible option in asymptomatic patients with <3 cm lesions and sequalae‐free resolution may be expected in least 20% of the cases.^2^, ^3^ Life‐long monitoring is recommended for complications and new lesions.^2^, ^3^

## AUTHOR CONTRIBUTIONS

**Serhat Kaya:** Writing – original draft; writing – review and editing. **Gamze Akkuzu:** Writing – original draft. **Ali Dablan:** Writing – original draft. **Cemal Bes:** Writing – original draft. **İlgin Özden:** Writing – original draft; writing – review and editing. **Aytül Hande Yardımcı** Supervision; writing – original draft; writing – review and editing.

## FUNDING INFORMATION

None.

## CONFLICT OF INTEREST STATEMENT

None declared.

## ETHICS STATEMENT

Not required by the institutional IRB due to the purely retrospective nature of the case report.

## CONSENT

Written informed consent was obtained from the patient to publish this report in accordance with the journal's patient consent policy.

## Data Availability

Data available on request due to privacy/ethical restrictions .
